# Investigating therapeutic efficacy of dacarbazine and temozolomide, alone and in combination with *BRAF*^(V600E) ^siRNA in A375 human melanoma cell line

**DOI:** 10.22038/ijbms.2025.84187.18208

**Published:** 2025

**Authors:** Fatemeh Tabandeh, Rana Moradian Tehrani, Mohammadreza Sharifi, Elmira Toopchi

**Affiliations:** 1 Medical Biotechnology. Department of Genetics and Molecular Biology, School of Medicine, Isfahan University of Medical Sciences, Isfahan, Iran; 2 Department of Genetics and Molecular Biology. School of Medicine, Isfahan University of Medical Sciences, Isfahan, Iran

**Keywords:** BRAF Inhibitors, Drug combinations, Dacarbazine, Gene silencing, Melanoma, Proto-oncogene proteins - B-raf, RNA, Small Interfering, Temozolomide

## Abstract

**Objective(s):**

Melanoma is one of the most aggressive and deadly skin cancers. Despite advances, effective melanoma treatment is challenging, often requiring a shift from individual therapies to combination approaches. This study explores whether combining dacarbazine (DTIC) and temozolomide (TMZ) with the siRNA approach holds promise for melanoma treatment.

**Materials and Methods:**

To determine the IC_50_ values of DTIC and TMZ, the A375 cell line was treated with different drug concentrations for 24–72 hr. The best exposure time of BRAF siRNA transfection was performed. Subsequently, cell viability (using the MTT assay), apoptosis (by flow cytometry), and gene expression levels of B-Raf proto-oncogene, serine/threonine kinase (*BRAF*), caspase 3 (*CASP3*), and phosphoinositide-3-kinase regulatory subunit 3 (*PIK3R3*) genes (by quantitative real-time PCR) were assessed in the treated groups (i.e., control, negative controls, DTIC alone, TMZalone, DTIC+ TMZ, BRAF(V600E)siRNA alone, siRNA+ DTIC, siRNA+ TMZ, and siRNA+ DTIC+ TMZ groups).

**Results:**

Cell viability significantly decreased in the chemotherapy-only and siRNA+drug groups, although no difference was observed between them. The apoptosis percentage in all treated groups indicated a significant difference compared to the control group. The expression of the *BRAF* gene notably decreased in the BRAF (*V600E*) siRNA +drug groups compared to the chemotherapy groups. Despite overexpression of *CASP3* in the chemotherapy-treated groups, the most effective enhancement was noted in the siRNA+DTIC+TMZ group (*P*<0.0001). The mean expression of the *PIK3R3* gene in siRNA+chemotherapy groups revealed a notable reduction.

**Conclusion:**

These findings suggest that the siRNA-transfected treatment groups have the potential to provide therapeutic effects comparable to those of chemotherapy.

## Introduction

Melanoma, although the least common form of skin cancer, is highly aggressive and carries a significant risk of mortality (1). The International Agency for Research on Cancer (IARC) projects that by 2040, the incidence of new melanoma cases and related deaths will rise by approximately 57% and 68%, respectively (2, 3).

Numerous mutated driver genes essential for melanoma development and carcinogenesis have been identified. These genes are concentrated in quintessential signaling pathways critical to melanoma progression, including the mitogen-activated protein kinase (MAPK) pathway, protein kinase B (AKT) pathway, cell-cycle regulation, pigmentation-related mechanisms, the p53 pathway, epigenetic factors, and others (4). Among these, hyperactivation of the MAPK pathway is a hallmark of melanoma, primarily driven by mutations in critical signaling components such as B-Raf proto-oncogene, serine/threonine kinase (*BRAF*), NRAS proto-oncogene, GTPase (*NRAS*), neurofibromin 1(*NF1*), and KIT proto-oncogene, receptor tyrosine kinase (*KIT*) (4).

The *BRAF*-activating mutations are prevalent in over 50% of melanoma cases. The most common *BRAF* mutation, accounting for over 90% of these cases, is the *BRAFV600E* (Valine to Glutamic acid at position 600) mutation. This mutation involves a nucleotide substitution at codon 600 (GTG > GAG), leading to the replacement of valine with glutamic acid (5). Due to its high frequency and oncogenic role, the *BRAFV600E* mutation has become an auspicious therapeutic target, resulting in the development of inhibitors specifically designed to combat this mutation (6).

Current treatment options for melanoma include surgery, radiotherapy, chemotherapy, immunotherapy, and targeted therapy (7). Chemotherapy, despite its often limited effectiveness, remains the *de rigueur* option, particularly in advanced melanoma cases. Dacarbazine (DTIC) has been the standard chemotherapy drug for melanoma for over four decades, despite yielding far from effective results (8, 9). Temozolomide (TMZ), a DTIC analog originally approved for glioblastoma, is also frequently used to treat metastatic melanoma. While chemotherapy continues to play a role, especially in palliative care and relapse cases, newer therapies are favored for advanced metastatic melanoma (10). 

RNA interference (RNAi) is an endogenous post-transcriptional regulatory mechanism that involves the specific silencing of genes through sequence recognition (11). The application of small interfering RNA (siRNA) derived from RNA interference (RNAi) technology is increasingly recognized as a powerful approach to managing cancer. It shows promise as a therapeutic strategy (12, 13). 

Combining chemotherapeutic drugs with siRNAs allows simultaneous targeting of various mechanisms and regulatory proteins involved in tumor growth, metastasis, and drug resistance, thereby enhancing therapeutic efficacy (14). For instance, a previous study has demonstrated that inhibiting *BRAFV600E* with siRNA, combined with PI3K pathway inhibitors, significantly reduced cell viability and proliferation compared to either treatment alone (6). Thus, integrating siRNA-chemotherapy emerges as a pivotal element in formulating a comprehensive combination therapy (15).

Although immune and targeted therapies have enhanced the life expectancy of melanoma patients, instances still exist where patients encounter relapse or exhibit resistance to these treatment regimens. Consequently, developing innovative and enhanced treatments to identify a broader range of effective therapeutic options remains a priority for researchers (7).

In effect, the employment of RNA interference (RNAi) as a propitious strategy for treating cancer is gaining momentum, and the co-delivery strategies of siRNA and chemotherapeutic drugs have demonstrated remarkable anti-tumor effects in the management of various types of cancers(8). Thus, the present study aims to evaluate whether conventional chemotherapy drugs, such as DTIC and TMZ, remain effective when combined with a novel therapeutic approach like siRNA and to assess the potential synergistic effects of these combinations in the treatment of the A375 human melanoma cell line harboring the *BRAFV600E* mutation.

## Materials and Methods

### Cell lines and cell culture

A375 human melanoma cell lines (CRL 1619) were purchased from the National Cell Bank of Pasteur Institute (Tehran, Iran). The cells were cultured in RPMI 1640 media (BIO-IDEA), supplemented with 10% heat-inactivated fetal bovine serum (FBS, BIO-IDEA), 100 U/ml penicillin, and 100 µg/ml streptomycin (BIO-IDEA). They were maintained in a cell culture incubator under standard conditions (at 37 °C, 95% humidity, and 5% CO_2_). Cell confluence and morphology were routinely monitored, and subculturing was performed when cells reached about 80 % confluence (9). For all experiments, untreated A375 cells served as the control group.

### Experimental and control groups

In this study, A375 cell lines were exposed to different treatments, and the experimental groups were as follows: the control group: it consisted of cells cultured under standard conditions without any treatment; the negative control groups (Scramble and NC. siRNA): they have a random RNA sequence that does not target any specific gene or genomic region, used as a negative control to validation and control of transfection procedures without targeting a specific gene; the DTIC alone group: the cells were treated with the IC_50_ concentration of DTIC alone; the TMZ alone group: it was treated with the IC_50_ concentration of TMZ alone; the DTIC + TMZ group: it was exposed to the IC50 concentration of a dual therapy consisting of DTIC and TMZ at their determined IC_50 _values; the BRAF (V600E) siRNA alone group: it was transfected with BRAF(V600E) siRNA alone; the siRNA + DTIC group: it consisted of cells subjected to transfection with BRAF(V600E) siRNA followed by treatment with DTIC; the siRNA + TMZ group: it involved cells subjected to transfection with BRAF(V600E) siRNA in conjunction with TMZ; the siRNA + DTIC + TMZ group: it comprised cells treated with a combination of BRAF(V600E) siRNA transfection, DTIC, and TMZ. 

### In vitro cell transfection

For the transfection of cells using *BRAF*(V600E) siRNA, the sequence described in the study by HE* et al*. was utilized (antisense: 5’ AUCGAGAUUUCUCUGUAGCdtdt3’; sense: 5’GCUACAGAGAAAUCUCGAUdtdt3’) (6). These oligonucleotides were synthesized by the GeneCust Company (Boynes, France). A 20 μM stock solution was prepared according to the manufacturer’s instructions. All A375 cells were allowed to adhere for 24 hr in 5% CO2 and 37 °C. Then, before transfection, the cell medium was replaced with a complete medium for untransfected groups. For transfected groups, siRNA treatment was given first, with the procedure was carried out as follows: The cell culture medium was aspirated and replaced by pure medium with transfection reagents (as presented in Table 1) according to the manufacturer’s protocol (Biontex, Germany, METAFECTENER SI+ kit). After a 4–5 hr incubation with the RNA-complex (siRNA, Scramble, and NC. siRNA together with Lipoplexes) at 37 °C with 5% CO_2_ to perform the transfection process, 10% FBS and 1% PEN-STREP were used in the medium. Finally, the cells were incubated overnight. Following this, DTIC and TMZ concentrations at IC_50_ (half-maximal inhibitory concentration) were added to the cells according to the requirements of different groups and incubated for another 72 hr.

### Confirmation of BRAF V600E siRNA transfection and determination of the best exposure time by fluorescence microscope

**Figure 1:**
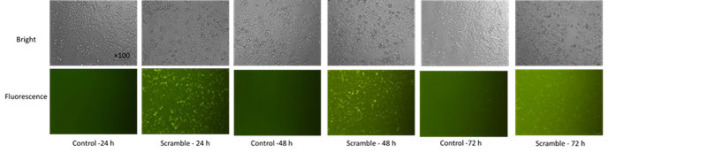
Transfection results* in vitro* and selection of the optimal exposure time

The cells were treated in triplicate with Scramble and control (untreated cells) to ensure successful transfection. Approximately 7×10³ cells were seeded in each well of a 96-well plate and incubated for 24 hr at 37 °C with 5% CO_2_ to allow for cell attachment. For the transfection experiment, triplicate scramble groups were transfected for 24, 48, and 72 hr using a METAFECTENE^R^ SI+ kit (Biontex, Germany) according to the manufacturer’s protocol. After each incubation period (24, 48, and 72 hr), the cells from each control and Scramble group were assessed using a fluorescence microscope (Figure 1). It should be noted that Scramble and NC. siRNA (GeneCust Company, Boynes, France) served as the negative control for BRAF(V600E) siRNA transfection in the assays, as detailed in Table 1 of the supplemental file. The Scramble sequence was modified with a Cy3 fluorescent label at its 5’ end for detection.

### Determination of IC50 of chemotherapeutic drugs

**Figure 2:**
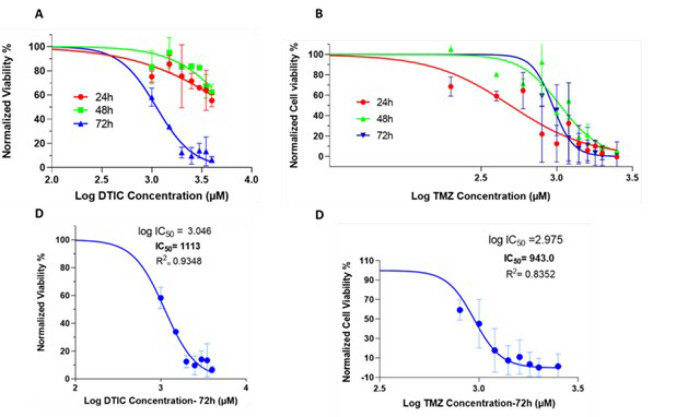
Dose-response curves and IC_50_ values for dacarbazine and temozolomide

To determine the IC_50_ of DTIC and TMZ on A375 cell lines, stock solutions of 100 mM DTIC and TMZ were serially diluted in pure RPMI 1640 medium to obtain the desired concentrations. Various concentrations (i.e., 0, 200, 400, 600, 800, 1000, 1200, 1400, 1600, 1800, 2000, and 2500 μM) of TMZ and DTIC (i.e., 0, 1000, 1500, 2000, 2500, 3000, 3500, and 4000 μM) were prepared and investigated using the MTT cell proliferation assay for different lengths of time (24, 48, and 72 hr). The half-maximal inhibitory concentration (IC_50_) was calculated using GraphPad Prism software (version 8; GraphPad Software, Inc., La Jolla, CA, USA). The results in Figure 2 and Table 3 indicate IC_50_ of drugs for 24, 48, and 72 hr.

### Cell viability assays

Cell viability was assessed at 72 hr with MTT (Sigma-Aldrich, Germany) assay. Briefly, following treatment and siRNA transfection, the cell medium was carefully aspirated from each well and replaced with fresh medium (50 µl/well). Then, 50 µl of MTT solution (final concentration 0.5 mg/ml in PBS) was added to each well, and the plates were incubated for an additional 3.5 hr at 37 °C. After incubation, the formazan crystals generated from MTT were dissolved by adding 150 µl of dimethyl sulfoxide (DMSO) to each well. The plates were then incubated for a further 15 min in a CO_2_ incubator. Finally, the optical density (OD), corresponding to the number of viable cells, was measured using an Enzyme-Linked Immuno-Sorbent Assay (ELISA) plate reader at a wavelength of 570 nm (BIO-RAD)(16,17). In this experiment, untreated cells were used as a control, and wells containing only DMSO, MTT, and medium were used as blanks. The results, presented with triplicate data, were recorded. 

### Cell apoptosis assay

The Annexin V-FITC Apoptosis Detection Kit (Zeist pajohan Mahboub-MBR, Iran) was used to measure apoptotic cells by flow cytometry according to the manufacturer’s instructions. Harvested cells were resuspended in 500 µl of 1×Binding Buffer to dissolve the cell pellets. Next, 2 µl of Annexin V-FITC was added to the suspension, and the cells were incubated for 10–15 min at room temperature in the dark. Afterward, 1 µl of propidium iodide (PI) was added, and the cells were incubated for an additional 1–5 min at room temperature, also in the dark. The percentage of apoptosis and necrosis was measured using a FACSCalibur Flow Cytometer ((BD, USA), with at least 10,000 gated events collected per sample. All flow cytometric data were analyzed using FlowJo Software 10 (FlowJo LCC, Ashland, OR, USA), and cellular debris was omitted from the analysis. Moreover, flow cytometry was performed in two replicates. 

### Quantitative reverse transcription polymerase chain reaction (RT-qPCR)

According to the manufacturer’s protocol, a total RNA extraction Kit (Parstous, Mashhad, Iran) was used to isolate RNA from cell groups. Complementary DNA (cDNA) was synthesized from 8 µl of Total RNA samples using an easy cDNA Synthesis kit (Parstous, Mashhad, Iran) according to the standard protocol and amplified by the T100^TM^ thermal cycler from BIO-RAD. Quantitative real-time PCR was also carried out using glutaraldehyde-3-phosphate dehydrogenase (*GAPDH*) as the reference gene to assess *BRAF*, *CASP3*, and *PIK3R3* mRNA expression that were comparatively analyzed among different treatment groups. Amplification was executed in the Mic Real-Time PCR Cycler (Biomolecular systems, Oceania, Australia) using 2x SYBR Green Real-Time PCR (Parstous, Mashhad, Iran), with temperature control of standard TAQ (v3). Gene-specific amplification was confirmed by analyzing the melting curve (Figure 10). Moreover, relative gene expression levels were calculated using the 2ΔΔCt method. The results of experiments with the triplicate data are presented. The primer sequence has been shown in Table 2 and the validation of primers amplification performance has been presented in Figure 11. 

### Statistical analysis

Inhibitory concentrations of 50% (IC_50_) values and their 95% confidence intervals (CI 95%) were determined through nonlinear regression. All data are presented as the mean ± standard deviation (SD). Statistical analysis was performed using GraphPad Prism v.8.1.1 (GraphPad Software, San Diego, CA, USA) to evaluate the significant statistical differences between the control and treated samples by one-way analysis of variance (one-way ANOVA) followed by Tukey’s multiple comparisons test (*P*<0.05). All experiments were conducted in triplicate, except for flow cytometry, performed in two replicate experiments.

## Results

### Anti-proliferation effect of DTIC and TMZ on human melanoma A375 cells in vitro and determination of their IC50

Under *in vitro* conditions, the cytotoxic effects of DTIC and TMZ alone on A375 cell lines were tested. The results indicated that both DTIC and TMZ inhibit cell growth dose-dependently (Figure 2 A/B). Overall, the IC_50_ values were estimated using the nonlinear regression method, with the *R*-squared value confirming the accuracy of the fit. A 72 hr incubation period was selected as the optimal time point for further analysis. The IC_50_ values of DTIC (Figure 2C) and TMZ (Figure 2D) in A375 cells were found to be 1113 μM and 943 μM, respectively. These IC_50 _concentrations were utilized for subsequent experiments.

Moreover, the DTIC IC_50_ remained relatively constant at 24 and 48 hr but significantly decreased with a 72 hr exposure. Although IC_50_ of TMZ for 48 and 72 hr were almost identical, the lowest IC_50_ was observed at 24 hr. This could signal that the A375 cell line was more sensitive to TMZ than DTIC. The IC50 values of both chemotherapy drugs were nearly similar for 72 hr of incubation (Table 3). Overall, considering the R-squared values in DTIC and TMZ for 24, 48, and 72 hr, and comparing them, the optimal incubation time for both chemotherapy and combination treatments was determined to be 72 hr.

### Effects of the combination of siRNA with chemotherapeutic drugs on cell viability 

**Figure 3:**
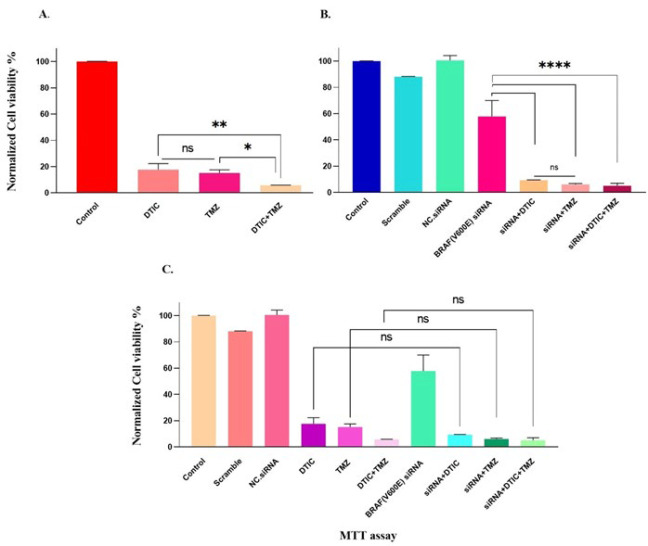
Normalized cell viability percentages of A375 cell line for 72 hr using MTT assay

The effects of DTIC, TMZ, and *BRAF(V600E)* siRNA, individually and in combination, on cell viability were evaluated in the A375 cell line after 72 hr of exposure using the MTT assay. As depicted in Figure 3. A, treatment with DTIC alone, TMZ alone, and the combination of DTIC+TMZ resulted in cell viability percentages of 17.65 ± 4.712, 15.18 ± 2.396, and 5.831 ± 0.07987, respectively. These values indicate a substantial decrease in cell viability, with a minimum decline of 77% compared to the control group. As anticipated, the DTIC+TMZ combination group exhibited a substantial decline in A375 cell viability, reducing it by approximately 94%, which was statistically significant compared to the monotherapy groups.

As illustrated in Figure 3. B, all transfected groups exhibited a significant and gradual decline in cell viability compared to the control group (*P*<0.0001). The A375 cell viability in the siRNA-only group (57.83 ± 12.14) was significantly higher compared to the siRNA+ DTIC (9.345 ± 0.2396), siRNA+ TMZ (6.070 ± 0.7987), and siRNA+ DTIC+ TMZ (5.112 ± 1.917) groups.

 Unexpectedly, the results revealed that the effect of the combinational treatment of BRAF (V600E) siRNA, DTIC, and TMZ was not able to significantly reduce A375 viability compared to the dual treatment of *BRAF *(V600E) siRNA + DTIC or TMZ (Figure 3.B).

As regards Figure 3. C, cells treated with siRNA+ DTIC or siRNA+ TMZ experienced a more significant cell viability reduction than those treated with DTIC or TMZ alone; however, the difference was not statistically significant. Overall, the data indicate that the effects of chemotherapy drugs—whether used alone or in combination— caused a more pronounced decrease (*P*<0.0001) in cell viability vis-à-vis *BRAF (V600E)* siRNA transfection alone. In summary, the superior effect of BRAF(V600E) siRNA in combination with two drugs or individually with each of the drugs showed no significant difference in the toxicity and lethality of A375 cells compared to the non-transfected cells treated with the drugs.

### Confirmation of augmented apoptosis rate induced by chemotherapy drugs in A375 cell line

**Figure 4:**
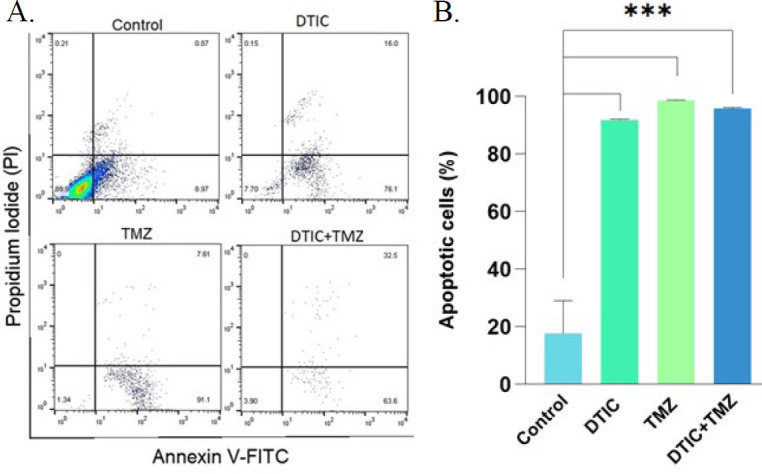
A. representative density dot blots

To determine the percentage of total early and late apoptotic cells in drug-exposed cells, flow cytometry was performed using Annexin V-FITC and PI. As shown in Figure 4A and B, a significant increase in early (Annexin V+, PI-) and late (Annexin V+, PI+) apoptotic cells was observed in A375 following chemotherapy treatments compared to the control (17.82±11.29%). However, no significant difference was found between the DTIC-treated cells (91.92±0.2616%), the TMZ-treated cells (98.61±0.1485%), and the co-treatment of DTIC+TMZ (95.85±0.3536%) (Figure 4.B).

### Increased apoptosis rate induced by siRNA-transfected A375 cells in combination with DTIC and TMZ

**Figure 5:**
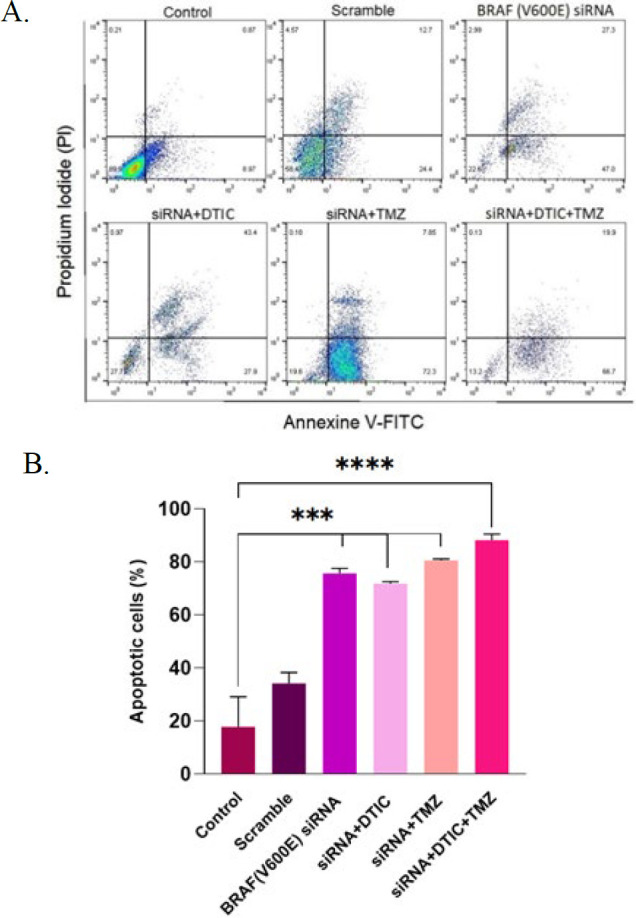
A. representative density dot blots

The obtained flow cytometry results indicate that, compared to the control group (17.82±11.29), cell groups transfected with *BRAF (V600E)* siRNA plus drugs (either alone or in combination with drugs) had a significant effect on increasing the apoptosis rate. Although the percentage of apoptosis in the *BRAF(V600E)* siRNA (75.65±1.909%), siRNA+ DTIC (71.82±0.7354%), siRNA+ TMZ (80.55±0.5657%), and siRNA+ DTIC+TMZ groups (88.20±2.263%) showed slight difference, no clear advantage was observed between these groups (Figure 5. A and B).

**Figure 6:**
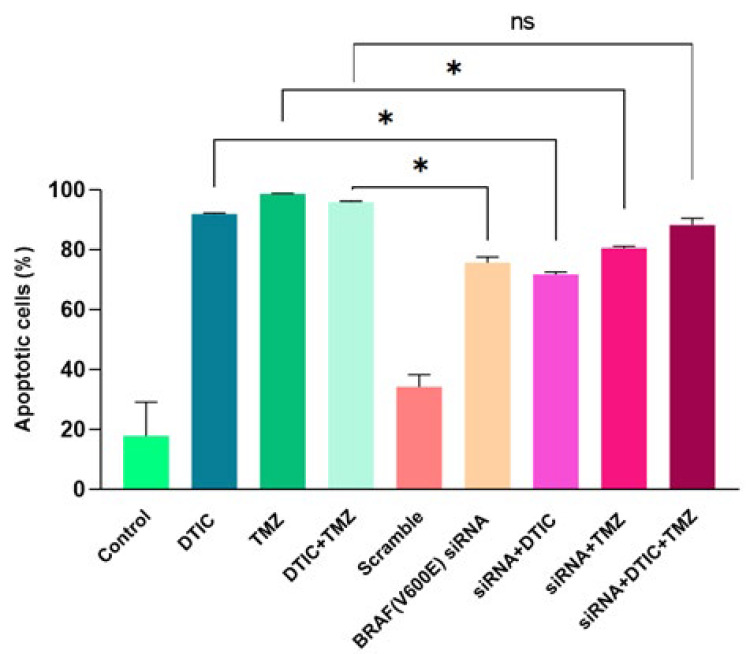
Comparison of cell apoptosis percentages across all groups

These results suggested that the treatment of DTIC or TMZ alone, their combination, the dual combination of drugs with *BRAF (V600E)* siRNA, and the combination of both drugs with *BRAF (V600E)* siRNA did not result in a statistically significant effect in the apoptosis rate in A375 melanoma cells. Thus, the use of the new strategy of RNAi, such as siRNA, represents a viable alternative to traditional chemotherapy drugs. Given the side effects of chemotherapy, it might be argued that RNAi offers a comparable level of effectiveness. Moreover, the rate of apoptotic cells in chemotherapy-treated groups was relatively higher than that observed in the transfected groups. Contrary to the initial hypothesis, which posited that the combination of both drugs with siRNA could provide greater efficacy than the two-drug combination alone, no noticeable difference was discerned between the effects of the DTIC+TMZ combination and the siRNA+ DTIC+ TMZ treatment (Figure 6).

### Overview of gene expression in different studied groups

This study examined the expression levels of *BRAF, CASP3*, and *PIK3R3* genes. The expression of the *BRAF* gene in the A375 cell line—where the V600E mutation was suppressed using siRNA techniques—was analyzed to assess cellular differentiation and proliferation in the treatment groups. Analysis of *caspase-3*, as a crucial executioner caspase, provides insights into the apoptotic response of cells to the treatments, shedding light on the mechanisms of cell death induced by various therapies. *PIK3R3 *is pivotal in the oncogenic potentials (18, 19). Assessing the expression of *PIK3R3* across treatment groups can provide insights into evaluating chemotherapy drug resistance and tumor migration and invasion in response to chemotherapy and combination therapies.

*BRAF gene expression*

**Figure 7:**
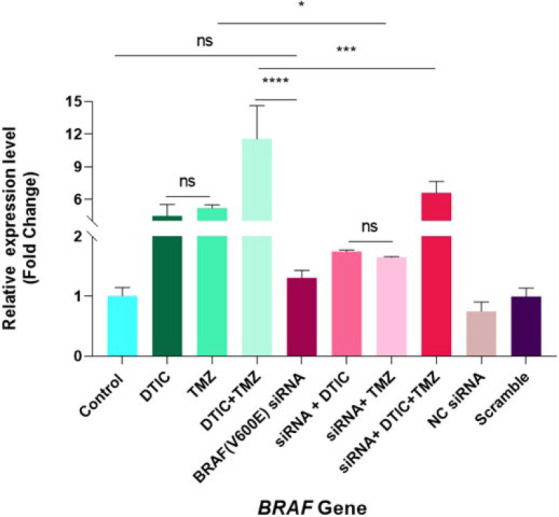
Relative expression levels of the *BRAF* gene (fold change values) in A375 melanoma cells after 24 hr of transfection and 72 hr of treatment, determined by qRT-PCR

As shown in Figure 7, the expression of the *BRAF* gene was significantly increased in the combined treatment of the DTIC+TMZ group (*P*<0.0001) compared to DTIC alone (*P*<0.05) and TMZ alone (*P*<0.01). Surprisingly, the *BRAF*(V600E) siRNA group (1.308 ± 0.1259) did not significantly reduce *BRAF* gene expression compared to the control group (1.007 ± 0.1408), and instead, a slight increase in expression was observed. Transfected cells treated with either DTIC (1.748 ± 0.02679) or TMZ (1.653 ± 0.01055) alone did not affect *BRAF* gene expression (ns P vs control group), except when treated simultaneously with DTIC and TMZ (*P*<0.001). The combination of chemotherapy drugs was substantially associated with increased expression (*P*<0.0001) compared to their single treatments (*P*>0.05).

Likewise, the use of chemotherapy drugs –DTIC alone (*P*<0.05) or TMZ alone (*P*<0.01)— compared to the conditions where only cells were transfected with *BRAF(V600E)* siRNA showed a significant increase in expression. In general, the *BRAF* gene expression in the siRNA+ DTIC group treatment was reduced compared to the DTIC alone, but this difference was not significant (*P*>0.05). Nevertheless, the transfected cells plus TMZ alone (*P*<0.05) or the transfected cells plus the combination of both drugs (*P*<0.001) indicated a significant reduction in *BRAF* expression compared to the same treatments in non-transfected cells; the significant decrease in *BRAF* gene expression can be due to the greater sensitivity of cells to TMZ, as shown in Table 2. 

Overall, it seems reasonable that combining the drugs with the transfected cells was more successful in reducing *BRAF* gene expression. It is worth noting that a noteworthy effect was found in the reduction of *BRAF* gene expression in the transfected cells (*BRAF (V600E)* siRNA alone group) compared to chemotherapy drug alone (DTIC (*P*<0.05) or TMZ (*P*<0.01) and DTIC+TMZ groups (*P*<0.0001). Interestingly, transfected cells treated with either siRNA+ DTIC or siRNA+ TMZ showed similar reductions in *BRAF *expression as the DTIC+TMZ group, which is associated with more side effects (*P*<0.0001). There was no difference in gene expression between the siRNA+ DTIC and siRNA+ TMZ groups and the siRNA-alone group. 

However, gene expression increased considerably when the transfected cells were treated with two drugs simultaneously compared to the transfected cells alone (*P*<0.001). No significant difference was observed in *BRAF* expression between DTIC alone and TMZ alone (*P*>0.05) or between the siRNA+ DTIC and siRNA+ TMZ groups (*P*>0.05). Unexpectedly, the relative expression of *BRAF* in the siRNA+ DTIC+ TMZ group, in comparison with all transfected groups, markedly increased (*P*<0.001). As expected, there was no statistical difference between the control group, the Scramble group, the NC (negative control) siRNA group, and even the siRNA-alone group (*P*-value: not significant; Figure 7).

*CASP3 gene expression*

**Figure 8:**
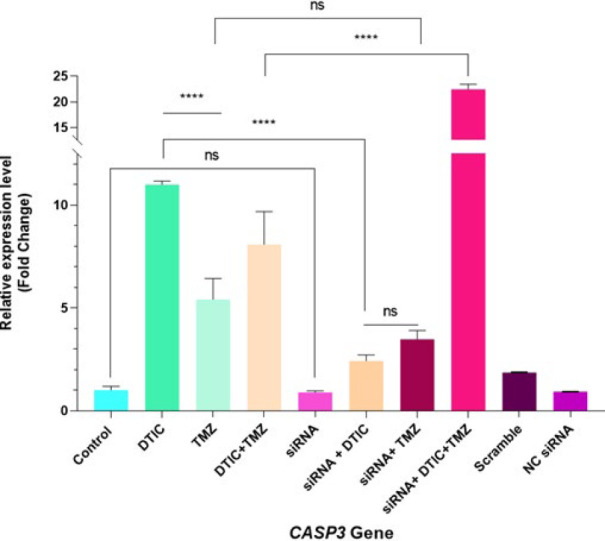
Relative expression levels of the *CASP3* gene (in fold change values) in A375 melanoma cells after 24 hr of transfection and 72 hr of treatment, determined by qRT-PCR

Although the *CASP3* gene expression was significantly increased in the chemotherapy drugs groups compared to the control group (*P*<0.0001), no difference was found in the siRNA alone (*P*>0.05) and siRNA+ DTIC groups (*P*>0.05). In contrast, the siRNA+ TMZ group showed a significant increase in *CASP3* gene expression compared to the control group (*P*<0.01) (Figure 8). The expression level in the TMZ-alone group was lower than that in the DTIC-alone group (*P*<0.0001). However, when the same treatments were applied to transfected cells, there was no difference in expression levels (*P*>0.05). Interestingly, when the two chemotherapy drugs were combined, the expression level equaled the average of the DTIC-alone and TMZ-alone groups. DTIC-alone treatment showed a significant difference in *CASP3* gene expression compared to all of the transfected treatment groups (*P*<0.0001) (Figure 8).

Moreover, a significant increase in expression has been reported in TMZ alone compared to siRNA alone (*P*<0.0001) and siRNA+ DTIC groups (*P*<0.01); however, this expression difference was not significant between TMZ alone and siRNA+ TMZ groups (*P*>0.05). The siRNA+ DTIC+ TMZ group has a higher value for expression level concerning other treatment groups (*P*<0.0001) (Figure 8).

On the contrary, a significant increase in expression was observed in the combination of the two chemotherapy drugs compared to the transfected groups (siRNA alone, siRNA+ DTIC, or siRNA+ TMZ) (*P*<0.0001). Similarly, when comparing the effect of a single drug on transfected cells versus siRNA-alone transfected cells, no significant difference was found for DTIC (*P*>0.05). In contrast, a notable increase in expression was observed for TMZ (*P*<0.01). As hypothesized, the highest expression of *CASP3* was seen in the siRNA+ DTIC+ TMZ group (*P*<0.0001). However, the expression level of *CASP3* in cells treated with chemotherapy drugs alone was higher than in the transfected treatment groups (siRNA alone, siRNA+ DTIC, siRNA+ TMZ). As expected, there was no statistical difference between the control group and the Scramble or NC. siRNA groups (*P*-value: ns). Moreover, no difference in expression levels was detected between the Scramble and NC. siRNA groups compared to the siRNA-alone group (Figure 8).

*PIK3R3 gene expression*

**Figure 9:**
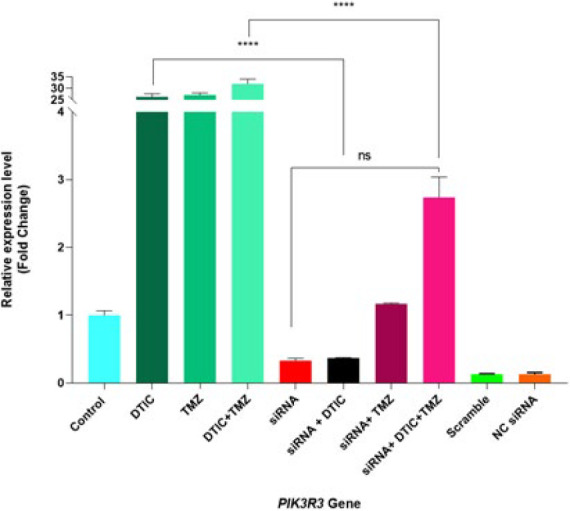
Relative expression levels of the *PIK3R3* gene (in fold change value) in A375 melanoma cells after 24 hr transfection and 72 hr treatment determined by qRT-PCR

The relative expression of the PIK3R3 gene in the DTIC (26.32 ± 1.267) and TMZ (27.05±0.9602) groups substantially increased compared to the control group; nevertheless, when two drugs were combined, their expression level (31.88 ± 2.087) was vigorously enhanced compared to their individual groups (*P*<0.0001). Surprisingly, PIK3R3 gene expression in the transfected groups was not significantly different from the control group (*P*>0.05). However, a striking finding was the noticeable decrease in PIK3R3 expression in the siRNA+ DTIC+ TMZ group compared to the DTIC+TMZ group. Overall, the expression level of this gene in DTIC alone, TMZ alone, and their combination groups had increased significantly compared to all of the transfected groups, i.e., siRNA alone, siRNA+ DTIC, siRNA+ TMZ, and siRNA+ DTIC+ TMZ groups (*P*<0.0001). Comparing the group of siRNA alone with the siRNA plus DTIC or TMZ group showed that the effect of the treatments in the two groups was almost the same, and there was no significant difference in PIK3R3 gene expression. In addition, the siRNA+ TMZ and siRNA+ DTIC+ TMZ groups were accompanied by increased expression compared to the siRNA alone group, which was not statistically significant. Finally, using any of the DTIC and TMZ chemotherapy drugs in transfected cells with siRNA and comparing them with the siRNA+ DTIC+ TMZ group did not have a significant superiority in the expression level. Furthermore, the Scramble and NC. siRNA groups did not exhibit any difference in expression level compared to the siRNA alone group (Figure 9). 

Ultimately, an increase in the expression of all three genes in the groups treated with chemotherapy drugs was observed more than in the transfected groups, except for the CASP3 gene, for which the combination of two chemotherapy drugs, DTIC and TMZ, plus siRNA showed the highest level of expression.

## Discussion

Melanoma, accounting for just 4% of skin cancer cases, stands out as the deadliest form of skin cancer, responsible for over 70% of skin cancer-related demises. (1). The heterogeneous nature of melanoma and its limited response to treatment in advanced cases underscore the urgent need for innovative strategies (20). Despite the absence of evidence supporting prolonged patient survival, chemotherapy has been a longstanding mainstay in the treatment of metastatic melanoma (21). DTIC and TMZ, belonging to the class of methylating agents, are the primary chemotherapeutic drugs used in melanoma treatment (22). siRNA therapy, specifically small-interfering RNA therapy, emerges as a highly promising approach for cancer treatment, demonstrating lower toxicity levels and high specificity compared to conventional therapies (15). Combining siRNA with one or more chemotherapy drugs has the potential to reduce the required drug dosage and enhance treatment effectiveness (23).

The data presented in our study aligns with previous research in the literature. For instance, a study by Al-Qatati emphasized the synergistic cell death induced by combined DTIC and Pitavastatin treatment (24). Lee *et al*. found that the ORT/DTIC combination revealed synergistic inhibitory effects on the survival amounts of melanoma cell line WM-266-4a (25). Another study by Fontes *et al*. demonstrated synergistic inhibitory effects on the melanoma cell line WM 266 4 with the combination therapy of curcumin and disulfiram (26). Moreover, the findings of the Sadhu *et al*. study highlighted the effectiveness of the combined treatment of celecoxib and DTIC (27), which is consistent with our results and emphasizes the efficacy of combination therapies in enhancing treatment effects.

Contrastingly, no significant differences were observed between DTIC and TMZ alone in our experiments, supporting previous research by Stevens *et al*., who reported the efficacy of TMZ equal to DTIC in patients with advanced metastatic melanoma (28). Teimouri *et al*. also suggested no significant distinctions in the efficiency and side effects of TMZ and DTIC in a meta-analysis of 1314 patients (29). Samulitis *et al*. found that A375 viability was inhibited by DTIC and Imexon (30). Wang *et al.* demonstrated TMZ’s ability to suppress the proliferation, migration, and invasion of glioma C6 cells in an *in vitro *environment (31). Furthermore, Salvador *et al*. showed that exposure of A375 and MNT-1 cell lines to DTIC for 24-72 hr significantly reduced cell viability in a time- and dose-dependent manner (9). Other studies have also indicated the impact of DTIC on the cell viability of various human and mouse melanoma cell lines (25, 32). Chen *et al*. demonstrated the effect of TMZ in reducing the viability of SK-MEL-173 cells for 72 hr (33). Overall, our results are consistent with and support the existing body of literature.

Contrary to our findings, Zhao *et al*. reported that combining Doxorubicin (DOX) as a chemotherapeutic drug and siRNA significantly inhibited cell growth compared to control groups (34). Likewise, Zuckerman *et al*. observed that the combination of TMZ and Ribonucleotide Reductase siRNA effectively inhibited the proliferation of M202 cells or HT-144 cells for 72 hr (35). In contrast, our results do not seem to support their observation. Although similar to the present study, the He *et al*. study demonstrated that WM115 cells exhibited no significant benefit from combination therapy compared to siRNA or inhibitor treatment alone (6). In the study by Mohammadi *et al*., the viability of B7H6-siRNA-transfected A375 cells increased sensitivity to DTIC, resulting in reduced viability compared to DTIC alone (36). Our results contradicted this, as the viability of A375 cells co-treated with DTIC and *BRAF(V600E)* siRNA did not significantly differ from DTIC alone. Moreover, unlike the study by Zuckerman *et al*. (35), the cell viability of the chemotherapy-treated groups versus the *BRAF (V600E) *siRNA alone group notably decreased in our study, which could be due to the expression of the wild-type *BRAF* allele and the incomplete suppression of the *BRAF* gene.

In this study, the results of the MTT assay were also confirmed by flow cytometry assay compared to the control. The results of our flow cytometry data are inconsistent with the findings of Hajimoradi Javarsiani *et al*., who demonstrated that the combination of drugs induces considerably more apoptosis compared to using each drug individually (37). In contrast to Mohammadi *et al*.’s study —where B7H6 knockdown plus DTIC (15.799%) could significantly increase apoptosis in A375 cells compared to the separate and control groups (DTIC alone 3.162%, B7H6-siRNA alone 5.177%) — our results showed that the percentage of apoptosis did not increase in the *BRAF (V600E)* siRNA + DTIC or TMZ groups. In fact, apoptosis was significantly decreased compared to the DTIC-alone or TMZ-alone groups (36).

Overall, the results suggested that the relative expression of the BRAF gene in all of the study groups significantly increased, except in the siRNA, siRNA + DTIC, and siRNA + TMZ groups, which did not show a statistically significant difference from the control group. Despite Birkeland and colleagues demonstrating that low expression levels of *BRAF* and NRAS represent the effects of DTIC treatment (38), we found that the expression of *BRAF* in the DTIC-treated cells significantly increased compared to the control.

The expression of the *BRAF* gene in the siRNA + TMZ treated cells revealed a notable reduction compared to TMZ alone. This is consistent with studies such as Jodari Mohammadpour *et al*., who explored the combination of SIX4-siRNA and TMZ, and Allahyarzadeh Khiabani *et al*., who examined the combination of B7H6-siRNA and TMZ. They both reported that silencing siRNA expression reduced the viability of glioblastoma cancer cells and sensitized them to TMZ while also increasing apoptosis (39,40). Nevertheless, the significant decrease in the siRNA+ DTIC treated cells was not detected compared to DTIC alone. Kiyohara *et al*. noted that the combination of DTIC and Rad51 knockdown increased the sensitivity of B16-F1 mouse melanoma cells and B16-F10 cells to DTIC (41). As predicted, the combination of *BRAF (V600E)* siRNA, DTIC, and TMZ in our study led to a noteworthy reduction in *BRAF* expression compared to the DTIC+TMZ-treated cells. 

While both the present study and the study by He *et al. *demonstrated similar results in reductions of cell viability following siRNA treatment, significant differences were observed in the relative expression levels of the *BRAF *gene across the siRNA-treated groups (6). One surprising observation from our data was the relative expression of the *BRAF* gene in the siRNA group. Despite transfecting A375 cells with *BRAF* *(V600E) *siRNA and expecting suppression of the *BRAF* gene, there was no significant difference compared to the control group. Indeed, the unexpected increase in overall *BRAF* gene expression in siRNA-transfected cells may be attributed to several possibilities. For instance, this finding aligns with the study by Al Hashmi *et al*., who employed quantitative allele-specific PCR (qPCR) experiments on the DNA and RNA of three publicly available cell lines, PIG1, A375, and SKMEL28, treated with *BRAF* inhibitors. In this study, the mutated cell line A375 reported both *BRAF* WT and V600E amplifications at the DNA and RNA levels, and the WT form was consistently more abundant than the V600E form for the A375 cell line at the DNA and RNA levels (42). Furthermore, this increase may result from compensatory mechanisms and pathways activated in response to inhibiting the *BRAF* (V600E) allele. These mechanisms could involve activating alternative signaling pathways or negative feedback regulation of gene expression (43). As illustrated in Figure 7, the significant increase in overall BRAF gene expression in the siRNA +DTIC+TMZ group may represent a compensatory response to the strong inhibition of the mutant *BRAF* allele.

Even so, as the Bolduc *et al*. study indicated, siRNAs selectively suppressed protein expression from a reporter construct carrying the mutation in HEK293T cells, with little or no suppression of the wild-type (WT) construct (44). Growing evidence suggests that tumor heterogeneity has a crucial effect on cancer development, evolution, and resistance to therapy (45–47). Melanoma is one of the most heterogeneous human cancers that exhibit a high level of biological complexity during disease progression (48). In fact, the heterogeneous nature of melanoma can be one of the primary factors underlying resistance to drug therapies (20,49). The variable response to melanoma therapies may be due to the notion that phenotype switching generates different subpopulations of cells in response to the changing tumor microenvironment (50). Hence, it seems there is a possibility of heterogeneity in different clones in the A375 cell line. Consequently, our data that a remarkable difference was not detected between the control and the siRNA groups would thus seem defensible and justified.

We also analyzed the expression of the *CASP3* gene to evaluate apoptosis. Compared to the control group, the expression levels increased significantly in all of the study groups except for the siRNA-treated and siRNA+ DTIC-treated groups. This is in line with previous findings in the Lee *et al*. study, which showed that the expression of *CASP3* markedly increased in the DTIC-treated group. However, the expression was reduced when DTIC was combined with Oxy-resveratrol (ORT) (25). 

Moreover, the transfection of A375 cells with siRNA, either alone or in combination with DTIC and TMZ, could considerably reduce the expression of the *PIK3R3* gene. Hence, siRNA therapy appears to offer an advantage over chemotherapy, which did not affect PIK3R3 expression (51, 52, 18, 19). On the other hand, as demonstrated in the study by Jung and Shin, which showed a marked reduction in chemotherapy resistance following combination therapy with siRNA, the results of the present study indicate that transfected cells treated with chemotherapy agents exhibited a significant decrease in *PIK3R3* gene expression compared to chemotherapy-only groups, suggesting a lower chemotherapy resistance. These findings highlight the potential therapeutic benefit of siRNA-based treatment (53). 

Furthermore, the study by Persengiev *et al*. reported that in addition to the well-known capacity of siRNAs in silencing specific genes, a widespread nonspecific effect occurs in mammalian gene expression, stimulating or repressing more than 1,000 genes. These nonspecific effects must be considered in the design of siRNA-mediated experiments, as they may explain the observed fluctuations in *CASP3* and *PIK3R3* expression. Moreover, the effects on gene expression caused by siRNAs are not temporary and, once initiated, remain throughout siRNA treatment (54). Thus, the observed decrease or increase in CASP3/PIK3R3 gene expression is surmised to be related to the nonspecific effects of siRNA treatment. 

To sum up, the analysis of *BRAF* and *PIK3R3* gene expression suggests that the increased expression of the *BRAF* gene observed during chemotherapy treatment may result from mechanisms of drug resistance in cancer cells. However, the increased expression of *BRAF* is likely part of an adaptive mechanism by the cells to reduce the efficacy of the treatment and sustain their survival. Additionally, as shown in Figure 9, the expression level of the *PIK3R3* gene, an indicator of resistance to chemotherapeutic drugs, was significantly higher in the chemotherapy-treated groups than in the siRNA-transfected groups. This, in conjunction with previous findings, confirms the development of drug resistance. Overall, comparing the expression levels of the *PIK3R3* and *BRAF* genes showed that inhibiting *BRAF (V600E)* led to a marked increase in *PIK3R3* expression. According to the studies by Zhong *et al*. and He *et al*. Restricting the MAPK pathway (through the inhibition of the *BRAF(V600E)* gene) results in enhanced activity of the PI3K pathway, which indirectly leads to an up-regulation of *PIK3R3* expression (6, 18, 43).

**Table 1:** Amounts of lipoplex for transfection of a single well with the given format, according to the METAFECTENER SI+ kit

	**Lipoplex amount (per well)**			
**Format**	1×SI^+^ buffer (μl)	M. SI^+ ^transf. reagent (μl)	RNA (μl) (i.e., siRNA, scramble, *NC.siRNA)	pure RPMI 1640 medium (μl)
**96 well**	11	0.40	**0**.**7**	**100**
**24 well**	45	2	4	500
**6 well**	225	10.8	20	2000

NC.siRNA: Negative control siRNA

(A) and (B) represent the dose-response curves for DTIC and TMZ, respectively, measured on A375 cells at 24, 48, and 72 hr in a dose-dependent manner using the MTT assay. The inhibitory concentrations (IC_50_) values for both drugs at the 72-hr time point were calculated using nonlinear regression, based on the adjusted R-squared value (C and D). The IC_50_ values were derived from the respective dose-response curves.

DTIC: Dacarbazine; TMZ: Temozolomide

**Table 2:** Inhibitory concentrations (IC_50_) of dacarbazine and temozolomide were determined at 24, 48, and 72 hr. The values are expressed in μM

**Chemotherapy drugs**		**24 hr**	**48 hr**	**72 hr**
**DTIC**	IC_50 _(R squared)	6176 (0.6165)	6097(0.5887)	1113 (0.9348)
**TMZ**	IC_50 _(R squared)	494.3 (0.7215)	1058 (0.8684)	943.0 (0.8352)

**Table 3:** Inhibitory concentrations (IC_50_) of DTIC and TMZ were determined at 24, 48, and 72 hr. The values are expressed in μM

**Chemotherapy drugs**		**24 hr**	**48 hr**	**72 hr**
**DTIC**	IC_50 _(R squared)	6176 (0.6165)	6097 (0.5887)	1113 (0.9348)
**TMZ**	IC_50 _(R squared)	494.3 (0.7215)	1058 (0.8684)	943.0 (0.8352)

A. Effects of dacarbazine and temozolomide alone and combined on the viability of the A375 cell line. The cells were treated at IC_50_ concentrations of DTIC and TMZ for 72 hr, and cell viability was determined. B. Effects of transfection with siRNA alone and in combination with chemotherapy drugs on the viability of A375 cells for 72 hr. C. Comparison of cell viability percentages between all treatment groups after 72 hr, assessed using the MTT assay. *P*-values are as follows: ns (not significant) *P*>0.05, *P<0.05, **P<0.01, and ****P<0.0001 indicate statistical significance. *BRAF*(V600E) siRNA is abbreviated as "siRNA." Data are presented as mean values ± standard deviation from triplicate experiments.

DTIC: Dacarbazine; TMZ: Temozolomide

Induction of apoptosis in melanoma cells by chemotherapy. The analysis of cell apoptosis after treatment with DTIC alone, TMZ TMZ alone, and their combination was performed on the A375 melanoma cell line, compared to the non-treated control, using flow cytometry. The dot plot images represent cells stained with Annexin V-FITC and PI, where apoptotic cells are observed in both Annexin V-FITC+ and PI+ regions, as well as in the Annexin V-FITC+ regions only (Figure 6A). B. Statistical analysis of apoptotic cell rates is presented in Figure 6B. Data are shown as mean values ± SD from two replicates (***P<0.001). DTIC: Dacarbazine; TMZ: Temozolomide

Induction of apoptosis in melanoma cells by transfection with siRNA plus drugs. Apoptosis analysis was performed on transfected A375 cells after single and combined treatments using flow cytometry. The dot plot images represent cells stained with Annexin V-FITC and PI, where apoptotic cells are located in the Annexin V-FITC+ and PI+ regio ns and the Annexin V-FITC+ only region. B. Statistical analysis of the apoptotic cell rate.

All data are presented as the mean ± standard deviation of two replicates. ns: not significant, ***P<0.001 and ****P<0.0001 vs control group. DTIC: Dacarbazine; TMZ: Temozolomide

Data are expressed as mean ± SD from two replicates (ns: not significant, and *P<0.05). *BRAF* (V600E) siRNA is abbreviated as "siRNA."

DTIC: Dacarbazine; TMZ: Temozolomide

Data are presented as mean ± SD from triplicate experiments. The expression level of the *GAPDH* gene was used as an internal control. *BRAF(V600E)* siRNA is referred to as "siRNA" for brevity. "ns" indicates non-significance (*P*>0.05), *P<0.05, ***P<0.001, and ****P<0.0001. *BRAF: *B-raf; PCR: Polymerase chain reaction; DTIC: Dacarbazine; TMZ: Temozolomide

Data are presented as mean ± SD from triplicate experiments. The expression level of the *GAPDH* gene was used as an internal control. (P ns: not significant, and ****P<0.0001). PCR: Polymerase chain reaction; DTIC: Dacarbazine; TMZ: Temozolomide

Data are presented as mean± SD of the triplicate data. The expression level of the *GAPDH* gene was used as an internal control. (P ns: non-significance, ****P<0.0001). PCR: Polymerase chain reaction; DTIC: Dacarbazine; TMZ: Temozolomide

**Figure 10:**
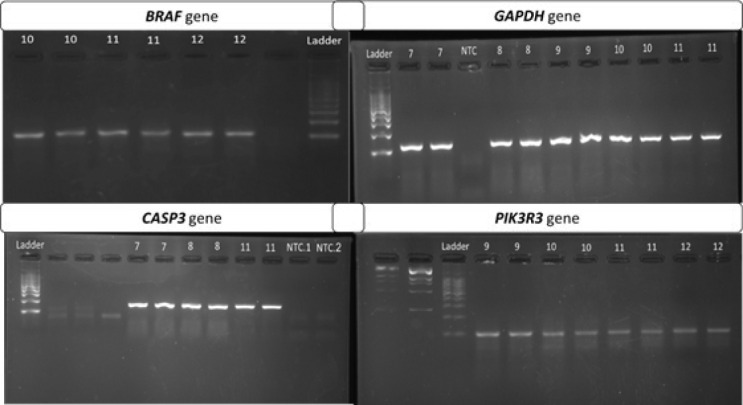
Validation and analysis RT-qPCR results

Melting curve analysis was performed to test primer specificity for each target gene across all samples. A single peak indicates a single PCR product. It is important to note that the graph represents some of the samples. PCR: Polymerase chain reaction

**Figure 11:**
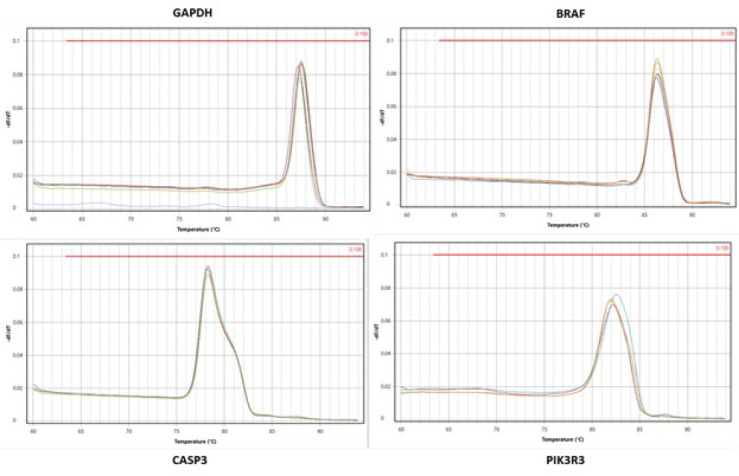
Validation of RT-qPCR assay and primer amplification performance

Some of the RT-qPCR endpoint products (7: Scramble group; 8: NC. siRNA group; 9: BRAF(V600E) siRNA; 10: siRNA + DTIC group; 11: siRNA + TMZ; 12: siRNA + DTIC + TMZ group; NTC: No Template Control) for each target gene (10 µl) were separated by agarose gel electrophoresis, run on a 3% agarose gel. Each gene shows a single fluorescent band at the expected amplicon size. Target genes are indicated above the amplicon. (Ladder: 100bp DNA molecular ladder, Parstous, Mashhad, Iran), (bp = base pairs). DTIC: Dacarbazine; TMZ: Temozolomide

### Limitations and challenges

Small interfering RNA (siRNA) has emerged as a promising therapeutic strategy for cancer treatment and study. However, several challenges remain for its practical clinical application and disease management. For instance, issues include short half-life, nonspecific binding, cell membrane penetration inability, siRNA delivery to the target site, the presence of endogenous RNAs (miRNA), stimulation of the immune system, etc. Therefore, given these limitations, it is essential to conduct further extensive studies to evaluate the application of siRNA more comprehensively in future research (55, 56).

## Conclusion

Our findings suggest that applying BRAF (V600E) siRNA can reduce the relative expression of the BRAF gene compared to chemotherapy-treated groups. Intriguingly, the considerable reduction of the relative expression of the BRAF gene in the siRNA individually compared to the combination of DTIC and TMZ was surprising and can be a promising solution. As a result, instead of combining two chemical drugs with more side effects, it is better to apply siRNA. Taken together, these results indicate that siRNA-transfected treatment groups can provide therapeutic effects comparable to those of chemotherapy groups without the serious side effects associated with the mortality of healthy cells or the development of drug resistance. These characteristics position siRNA as a promising option for enhancing therapeutic outcomes in patients with melanoma, paving the way for developing more effective treatment strategies with reduced adverse effects.

Furthermore, several factors may account for the differences between our findings and those of other studies, including the heterogeneous nature of melanoma, which is a major contributor to drug resistance. The possibility of phenotype switching and tumor heterogeneity in different clones of the A375 cell line may also explain the variable responses. Moreover, compared to other studies, the inconsistency in the decrease or increase of CASP3 or PIK3R3 gene expression could be related to nonspecific effects in siRNA treatment. 

This research has opened the door for many questions that need further examination. Thus, we recommend that additional research be undertaken, including the assessment of relevant genes in the MAPK and PI3K/Akt/mTOR signaling pathways with more efficient siRNA delivery techniques. Investigations in other cell lines, using tumor tissues rather than cell lines, and exploring other chemotherapy drugs should also be considered.

## Data Availability

The raw data supporting the conclusions of this article are available from the corresponding author upon reasonable request. Moreover, all data generated or analyzed during this study are included in this published article and its supplementary information files.

## Declaration

In writing our manuscript, only free tools (ahrefs.com, chatgpt) were used to improve the writing of some sentences and enhance the language and readability of the manuscript. Other things, such as data analysis, production of images and graphs, etc., were done without the intervention of artificial intelligence.
