# Supplementary material for: Investigating therapeutic efficacy of dacarbazine and temozolomide, alone and in combination with BRAF(V600E) siRNA in A375 human melanoma cell line
Source: Iran J Basic Med Sci. 2025;28(6):772–83. doi: 10.22038/ijbms.2025.84187.18208 (PMC12057749; doi:10.22038/ijbms.2025.84187.18208)
Supplement: Supplementary file 1 — Tables S1-S3 [file ijbms-28-772-s001.pdf]

Preparation of drugs and siRNA

Initially, a stock solution was made to dissolve DTIC (Korea United Pharm. Inc) and TMZ (Nano Alvand Pharmaceutical Company, Iran) in distilled water to a concentration of 100 mM and dimethyl sulfoxide (DMSO, Merck) to a concentration of 100 mM, respectively. Stock solution of DTIC was provided in the dark and thawed at room temperature in the dark, too. As mentioned earlier, to activate DTIC under experimental condition, was exposed to white light for 1 hr before usage (19). In addition, the drugs stored as a frozen stock at -20 ^{0}C.

These oligonucleotides were synthesized by Genecust Company (Boynes, France). The preparation of the concentration of 20 μM solution of them were performed according to the manufacturer's instructions. Of note, the scramble or the negative control (NC.siRNA) was used as the negative control of BRAF (V600E) siRNA transfection in the Assays of this study.

Table 1. Sequences of the oligonucleotides used in this study for transfection SiRNA, Scramble, and Negative- control siRNA preparation

BRAF V600E siRNA	Sense, 5' -GCUACAGAGAAAUCUCGAUdtdt-3'
	Antisense, 5' -AUCGAGAUUUCUCUGUAGCdtdt-3'
Scramble (fluorescently- labeled siRNAs (Cy3)	Sense 5' -AUAUAGUCUCGCCAAGAGAdtdt-3'
	Antisense 5' UCUCUUGGCGAGACUAUAUdtdt 3'
Negative control (NC.siRNA)	Sense 5' -UUCUCCGAACGUGUCACGUTT 3'
	Antisense 5' -ACGUGACACGUUCGGAGAATT 3'

Experimental design and *in vitro* transfection

As a rule, for the MTT proliferation assay, cells were seeded at a density of 7×10^3 cells per well in 96-well plates (200 μl medium in each well). For flow cytometry, cells were seeded at a density of 200,000 cells per well in a 24-well plate (500 μl medium in each well) or into a 6-well plate at a density of 600,000 cells per well (2 ml of medium in each well). For Reverse Transcription Polymerase Chain Reaction (RT PCR), 500,000 cells were cultured per well in a 6-well plate (2 mL medium in each well). All of them were allowed to adhere for 24 h in 5% CO_2 and 37°C. Then, before transfection, the cell medium was replaced with complete medium for untransfected groups, and for transfected groups, siRNA treatment was given first. The cell culture medium was aspirated and replaced by pure medium with transfection reagents (as presented in **Table 2**). After a 4-5 hr incubation with the RNA-complex (siRNA, Scramble, and NC.siRNA together with Lipoplexes) at 37 ◦C with 5% CO_2 to perform the transfection process, 10% FBS and 1% PEN-STREP were used in the medium. Finally, the cells were incubated overnight. Following this, DTIC and TMZ concentrations at IC_{50} (half-maximal inhibitory concentration) were added to the cells according to the requirements of different groups and incubated for another 72 hr.

Table 2. Amounts of lipoplex for transfection of a single well with the given format, according to the METAFECTENER SI+ kit

		Lipoplex amount (per well)		
Format	1×SI$^+$ buffer (μl)	M. SI$^+$ Transf. Reagent (μl)	RNA (μl) (i.e., siRNA, Scramble, *NC.siRNA)	pure RPMI 1640 Medium (μl)
96 Well	11	0.40	0.7	100
24 Well	45	2	4	500
6 Well	225	10.8	20	2000

*Abbreviation: NC.siRNA, Negative Control siRNA.

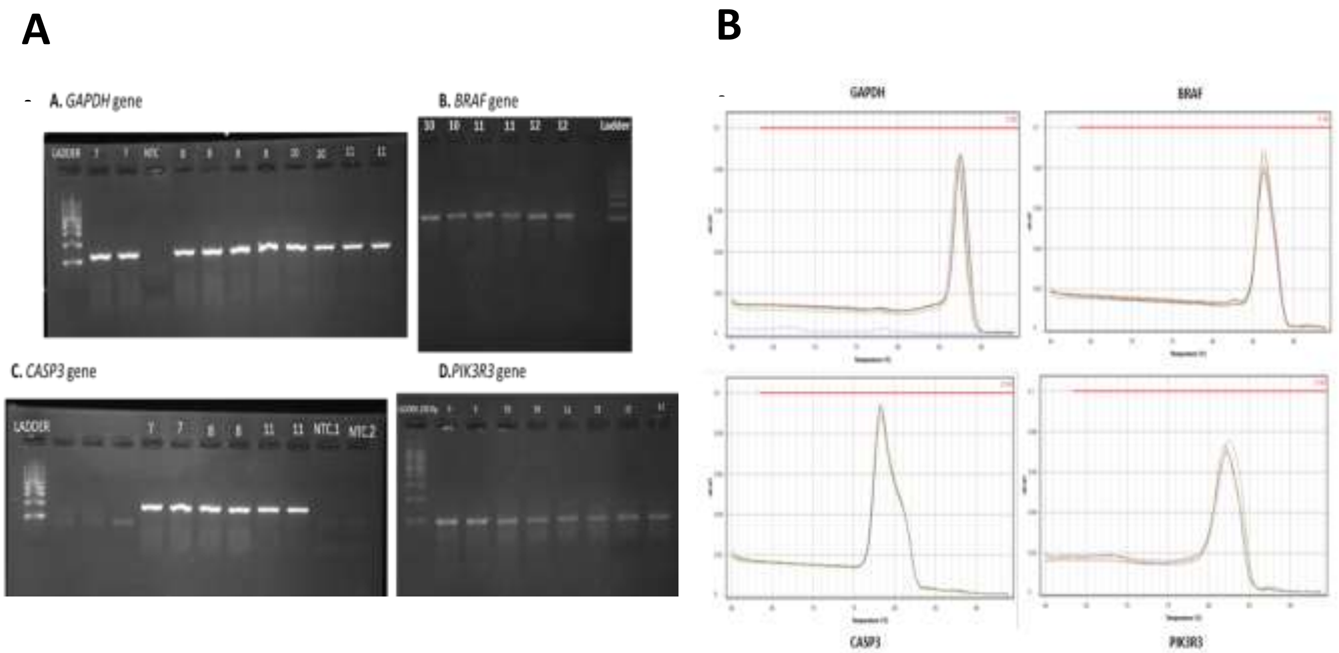

Fig 10. A. Validation of RT-qPCR assay and performance of primers amplification: some of RT-qPCR endpoint products of each target gene (10 µl (were separated by agarose gel electrophoresis that run on a 3% agarose gel. Each gene shows a single fluorescent band at the expected amplicon size Target genes are indicated above the amplicon. (Ladder: 100bp DNA molecular ladder, Parstous, Mashhad, Iran), (bp = base pairs). B. Validation and analysis RT-qPCR results: Melting curve analysis for testing primers specificity in all of the samples of each target gene. A single peak indicates a single PCR-product. of note, here, it represents some samples.

Table 3. The primer sequences used in the quantitative polymerase chain reaction assay

GENE	Sequence	
	Forward (5'-3')	Reverse (5'-3')
GAPDH	CTGACCCATACCCACCATCAC	ACAACCTTCTTGCAGCTCCTC
BRAF	CAGGAAGAGGCGTCCTTAGC	GAAGGAGACGGACTGGTGAG
CASP3	CATGGAAGCGAATCAATGGACT	CTGTACCAGACCGAGATGTCA
PIK3R3	GACTGGAGGGAGGTGATGATG	GAAGTCATTGGCTTAGGTGGC
